# Comprehensive characterization reveals sputum supernatant as a valuable alternative liquid biopsy for genome profiling in advanced non-small cell lung cancer

**DOI:** 10.1186/s12931-022-02097-4

**Published:** 2022-07-01

**Authors:** Xiaohong Xie, Jianhui Wu, Bingpeng Guo, Liqiang Wang, Haiyi Deng, Xinqing Lin, Ming Liu, Yinyin Qin, Wei Luo, Yilin Yang, Xiao Zou, Ting Hou, Jianxing Xiang, Zhange Chen, Chengzhi Zhou

**Affiliations:** 1 State Key Laboratory of Respiratory Disease, National Clinical Research Center for Respiratory Disease, Guangzhou Institute of Respiratory Health, The First Affiliated Hospital of Guangzhou Medical University, 151 Yanjiangxi Road, Guangzhou, 510120 Guangdong China; 2 Burning Rock Biotech, Room 601, Building 6, Phase 2, Standard Industrial Unit, No. 7 LuoXuan 4th Road, International Biotech Island, Guangzhou, 510300 Guangdong China

**Keywords:** NSCLC, Liquid biopsy, Sputum supernatant, Driver alterations, Genomic profiling, Next-generation sequencing

## Abstract

**Background:**

Sputum biopsies offer unique advantages such as non-invasiveness and convenient collection. The one investigation so far on sputum for genome profiling in advanced non-small cell lung cancer (aNSCLC) suggested promising performance. However, it remains undefined whether clinicohistologic characteristics were associated with performance and how this knowledge could help guide choice of liquid biopsy.

**Methods:**

Targeted sequencing with a 520-gene panel was performed on prospectively collected matched tumor tissue (TIS), plasma (PLA), and sputum supernatant (SPU) from 71 aNSCLC patients (NCT05034445). Genomic alteration detection was characterized in a series of aspects and interrogated for association with 14 clinicohistologic features. Nomograms were constructed with logistic regression for predicting the liquid biopsy type with greater sensitivity.

**Results:**

Compared with PLA, SPU showed comparable quality control metrics, mutation detection rate (SPU: 67.6%, PLA: 70.4%), concordance with tumor tissue (67.6% vs. 73.2%), and correlation with tissue-based tumor mutation burden levels (*r* = 0.92 vs. 0.94). For driver alterations, detection was less sensitive with SPU (50.0%) than PLA (63.5%) in the entire cohort but similarly or more sensitive in patients with centrally located lung tumors or smoking history or for altered *ALK* or *KRAS*. Two nomograms were constructed and enabled predicting the probability of superior sensitivity with SPU with moderate to borderline high accuracy.

**Conclusion:**

In addition to demonstrating comparable performance in multiple aspects, this study is the first to propose nomograms for choosing liquid biopsy based on clinicohistologic characteristics. Future research is warranted to delineate the clinical utility of sputum for genome profiling.

**Supplementary Information:**

The online version contains supplementary material available at 10.1186/s12931-022-02097-4.

## Introduction

Tumor and blood currently constitute the major materials for molecular testing in advanced non-small cell lung cancer (aNSCLC) [1]. Meanwhile, alternative liquid biopsies are actively pursued, as they may be even less invasive and more readily available (e.g. sputum and urine) or supplement the blood or even outperform it for detecting genomic aberrations in some patient populations. So far, there is evidence demonstrating the feasibility of genotyping with fluids that may contain neoplastic cells, such as malignant pleural effusions, cerebrospinal fluid, urine, and sputum [2–4]. Specifically, four studies focused on sputum as an alternative to blood for mutation analysis [3, 5–7]. However, most of these investigations focused on detecting sensitizing mutations and T790M mutation in epidermal growth factor receptor (*EGFR*) in aNSCLC, typically using polymerase chain reaction (PCR)-based technologies [5–7]. Currently only study reported on the performance of sputum for genomic profiling with targeted gene panels [3]. NSCLC-related driver alterations were detected with cell-free DNA (cfDNA) extracted from plasma and sputum from 50 aNSCLC patients using a 416-gene panel. When compared against those from matched tumor samples, concordance rates were 86% for plasma and 74% for sputum. Moreover, smokers showed higher tissue-sputum concordance than non-smokers [3]. These findings demonstrated comparable performance for plasma and sputum in terms of driver alteration detection and suggested promise for clinical applications involving sputum [8]. Further questions follow, such as other clinical characteristics of patients with more concordant detections, the feasibility of using these characteristics to guide the choice of liquid biopsy in profiling beyond driver alterations, and tumor mutation burden (TMB) estimation using sputum. There is therefore a gap in our understanding of the clinical utility of sputum for genomic profiling in aNSCLC.

This study aims to address this gap by characterizing the mutation detection from sputum samples in more aspects. We enrolled 71 aNSCLC patients, performed genome profiling on matched tumor, sputum and plasma, and compare their performance in mutation detection. A series of clinicohistologic characteristics were interrogated for association with improved detection of genomic alterations in all targeted genes as well as all and each of NSCLC-associated drivers. Nanograms were then constructed to help choose between sputum and plasma when tumor biopsies are not available.

## Patients and methods

### Patients

This prospective study enrolled 71 treatment-naïve stage III-IV NSCLC patients at The First Affiliated Hospital of Guangzhou Medical University from Sep. 2019 to Sep. 2020 (NCT05034445). The study was approved by the hospital’s Ethics Committee and written informed consent was obtained from all patients or corresponding family members. Clinicohistologic features were retrieved from the patients’ medical records, among which centrally located lung tumors were defined as within 2 cm of the proximal bronchial tree and/or abutting mediastina pleura.

### Sample collection and pre-processing

Matched tumor, peripheral venous blood (8 ml), and sputum (2 ml) were collected from same patient before the first-line therapy. Induced sputum was collected in case a patient did not produce spontaneous sputum for the first 45 patients, and for the remaining 26 patients only spontaneous sputum was collected due to limitations in hospital visits since the COVID-19 pandemic. A total of 13 induced sputum samples were collected. Specifically, forced expiratory volume (FEV_1_) was measured by spirometry before and ten minutes after inhalation of 400 µg albuterol. Sputum induction was performed with 4.5% saline inhalation for 15 min for patients with FEV_1_ ≥ 1L and 0.9% saline for those with FEV_1_ < 1L.

Sputum samples were mixed with equal volume of fixing buffer and then treated with 0.25% pancreatin for 30 min at 37 °C at a maximum of 1:2.5 sputum to pancreatin ratio until complete liquefaction. After centrifugation at 3000*g* for 10 min at 4 ℃, the supernatant was subjected to another centrifugation at 16,000 × g for 10 min at 4 ℃, aliquoted, and stored at − 80 ℃. Blood was collected in a standard ethylenediaminetetraacetic acid tube and subjected to routine processing described in previous studies [9]. All procedures were finished within two hours of sample collection.

### DNA extraction, library construction, targeted sequencing, and bioinformatic analysis

All wet-lab procedures were performed at a clinical diagnostic laboratory certified by both the College of American Pathologists and Clinical Laboratory Improvement Amendments. Briefly, DNA was extracted from formalin-fixed, paraffin-embedded tissue samples with a QIAamp DNA tissue kit, and cfDNA with a QIAamp Circulating Nucleic Acid kit (Qiagen, Düsseldorf, Germany). DNA library construction, and targeted sequencing with a commercial panel of 520 cancer-related genes (Burning Rock Biotech, Guangzhou, China) were performed as previously described [2, 9]. Sequencing was conducted on Nextseq500 (Illumina, San Diego, USA) at a target depth of 1000 × for tumor samples and 10,000 × for plasma or sputum samples. Bioinformatic analysis were performed as previously described [2, 9]. Tumor mutation burden (TMB) was calculated as number of non-synonymous somatic alterations on the coding regions of the targeted genes per million base pairs after excluding variants with allelic frequency < 2% from tissue samples or < 0.2% from liquid biopsy samples.

### Nomogram construction and validation

The predictive model was developed with multivariate logistic regression analysis in a stepwise fashion using the R version 4.0.2 (https://cran.r-project.org/) and extension package named rms. Multivariate logistic regression analysis was performed to identify the clinicohistologic features correlated with more sensitive detection with SPU than with PLA. Predictive models were established with these features and subjected to stepwise (stepAIC) selection to arrive at the best possible model based on Akaike Information Criteria (AIC). A nomogram based on the predictive model was developed. The discriminatory ability of the predictive model was validated using the receiver operating characteristic curve (ROC) and leave-one-out method. The nomogram models were internally validated with tenfold cross-validation. A total of 200 repetitions of tenfold cross-validation were performed and area under curve of the resulting ROC curves were computed.

### Statistical analyses

Mutation detection rate was defined as the ratio of the number of samples detected with any somatic mutation to the total number of samples of the same type. True positive patients were defined as those who carried at least one genomic alteration that were detected in both matched samples, and patients were as defined true negative if no alteration was detected from either matched sample. Concordance rate was defined as the fraction of the total number of true positive and true negative patients relative to the cohort or indicated subgroup. Maximum allelic fraction was defined as the maximum fraction of the mutant allele detected by NGS from a sample. Fisher’s exact test was used to compare the proportions of values of a nominal variable between two groups. Wilcoxon signed-rank test was used to compare the central tendency of a continuous variable between two groups. All statistical analyses were performed using the R programming language. Significance was set at two-side p < 0.05.

## Results

### Patient characteristics

This study enrolled 71 previously untreated patients with stage III-IV NSCLC. As indicated in Table 1, the cohort had a median age of 60 (range 20–79) with a majority of men (76.1%, n = 54). Fifty-six patients had adenocarcinoma (LUAD; 78.9%) and the remaining 15 had squamous cell carcinoma (LUSC; 21.1%). Most patients had metastatic disease (67.6%, n = 48), and there were approximately equal number of smokers and non-smokers (33 and 38 patients, respectively). Slightly more than half (54.9%, n = 39) of the cohort had a centrally located lung tumor, which was defined as one within 2 cm of the proximal bronchial tree and/or abutting mediastina pleura. Additionally, the majority patients did not present with tracheal violation (62.0%, n = 44), blood vessel invasion (78.9%, n = 56), or pleurisy (67.6%, n = 48), respectively.

**Table 1:** Baseline demographic and clinicohistologic characteristics of the patients described in this study

Clinicohistologic feature		n (%)
Age, median [range]	60 [20–79]	
Sex	Female	16 (22.5)
	Male	54 (76.1)
	Unknown	1 (1.4)
Stage	III	22 (31)
	IV	48 (67.6)
	Unknown	1 (1.4)
Histology	LUAD	56 (78.9)
	LUSC	15 (21.1)
Smoking	Yes	33 (46.5)
	No	38 (53.5)
Location of lung tumor	Central	39 (54.9)
	Peripheral	28 (39.4)
	Unknown	4 (5.6)
Sputum type	Induced	13 (18.3)
	Non-induced	58 (81.7)
T stage	T1	9 (12.7)
	T2	19 (26.8)
	T3	15 (21.1)
	T4	28 (39.4)
N stage	N0	1 (1.4)
	N1	7 (9.9)
	N2	22 (31)
	N3	40 (56.3)
	Unknown	1 (1.4)
M stage	M0	19 (26.8)
	M1	48 (67.6)
	Unknown	4 (5.6)
Nodal metastasis	Yes	68 (95.8)
	No	2 (2.8)
	Unknown	1 (1.4)
Brain metastasis	Yes	12 (16.9)
	No	58 (81.7)
	Unknown	1 (1.4)
Pleural metastasis	Yes	27 (38.0)
	No	43 (60.6)
	Unknown	1 (1.4)
Bone metastasis	Yes	12 (16.9)
	No	58 (81.7)
	Unknown	1 (1.4)
Tracheal violation	Yes	23 (32.4)
	No	44 (62.0)
	Unknown	4 (5.6)
Blood vessel invasion	Yes	12 (16.9)
	No	56 (78.9)
	Unknown	3 (4.2)
Pleurisy	Yes	19 (26.8)
	No	48 (67.6)
	Unknown	4 (5.6)

*LUAD* lung adenocarcinoma, *LUSC* lung squamous cell carcinoma

### Quality control (QC) metrics for mutation profiling using tumor tissue, sputum and plasma biopsies

**Fig. 1:**
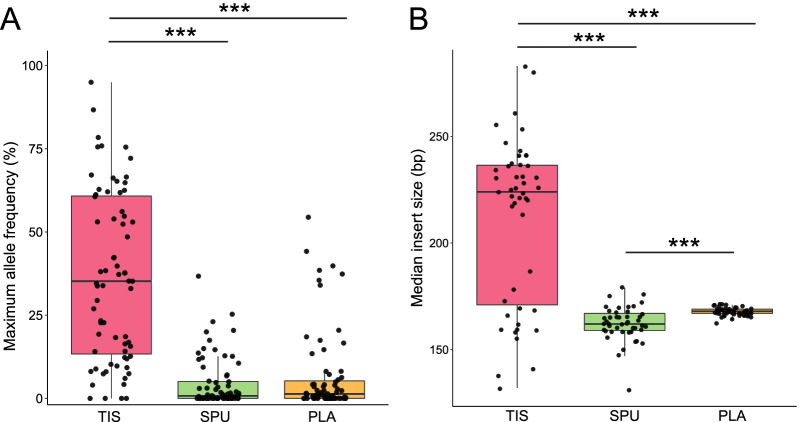
Distribution of key quality control indices for detected genomic alterations from biopsy samples of tumor (TIS), sputum supernatant (SPU), and plasma (PLA). **A** Distribution of maximum allele frequencies (maxAFs) among the three types of biopsy samples. Copy number variations, gene fusions, and other large genomic rearrangements were excluded from maxAF calculation. **B** Distribution of insert sizes. ***, p < 0.001

Next-generation sequencing was successfully performed on matched tumor tissue (TIS), sputum supernatant (SPU), and plasma (PLA) samples from all 71 enrolled patients. We started characterizing the performance of SPU samples by comparing important QC indices. In terms of DNA yield, SPU samples led to the highest yield with median of 2185 ng per sample, followed by TIS (median 560 ng) and PLA at significantly lower levels (median 50.5 ng). The liquid biopsy samples led to similar maximum allele frequencies (maxAFs), with median maxAF of 0.76% for SPU and 1.34% for PLA (p = 0.34), both of which significantly lower than TIS-derived maxAFs, which had a median of 35.2% (p < 0.001 for both pairwise comparisons; Fig. 1A). Distribution of insert sizes followed the same pattern. Inserts detected from SPU (median 162 base pairs [bp]) were slightly but significantly shorter than those from PLA (median 168 bp, p < 0.001). Both were significantly shorter than those from TIS samples (median 224 bp) by a considerable margin (p < 0.001; Fig. 1B). Collectively, these results suggest higher DNA yield from sputum supernatant and comparable maxAF and insert sizes when compared with plasma.

### Tumor mutation burden, overall detection rates, and clinicohistologic features associated with improved detection from sputum supernatant

**Fig. 2:**
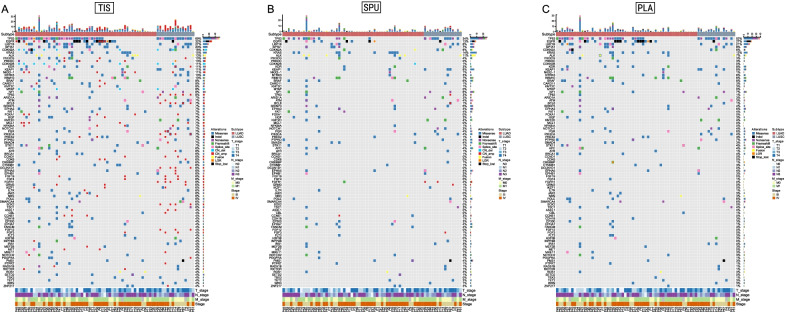
Representative profiles of the most frequently detected genomic alterations form **A** tumor tissue (TIS), **B** sputum supernatant (SPU), and **C** plasma (PLA) samples. These profiles show genes whose alterations were detected in ≥ 1 TIS and ≥ 4 SPU or PLA samples

**Fig. 3:**
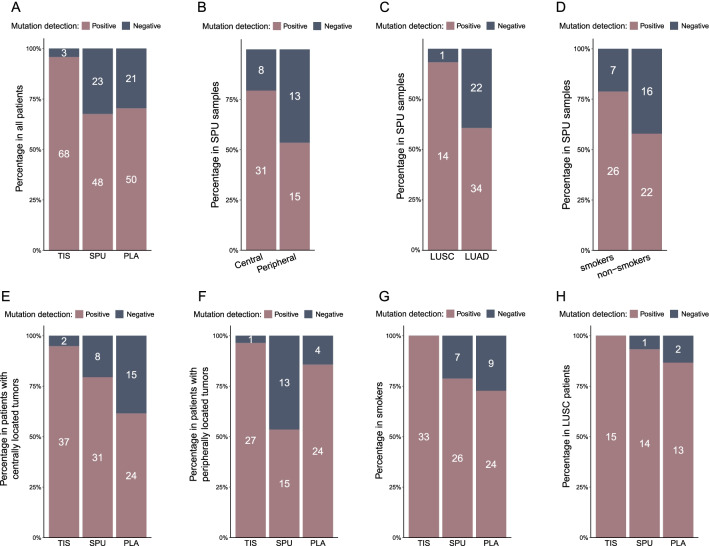
Rates of overall detection in the entire cohort or in specific patient subgroups. **A** Mutation detection rates of tumor tissue (TIS), sputum supernatant (SPU), and plasma (PLA) samples in all patients. Mutation detection rates of SPU among **B** patients with centrally vs. peripherally located lung tumors, **C** lung squamous cell carcinoma (LUSC) vs. lung adenocarcinoma (LUAD) patients, and **D** smokers vs. non-smokers. Comparison of detection rates of the three biopsy types in **E** and **F** patients with centrally or peripherally located lung tumors, respectively, **G** smokers, and **I** LUSC patients. Mutation detection rate was defined as the ratio of the number of samples detected with somatic mutations to the total number of samples of the same type. Numbers within the bars indicate the numbers of samples of the corresponding type, subgroup, and detection status

Figure 2 shows representative profiles of the most frequently detected alterations from each sample type. Both liquid biopsies showed strongly correlated tumor mutation burden (TMB) levels with tumor tissue samples and between themselves, as indicated by high Pearson correlation coefficients (SPU-TIS: 0.92, PLA-TIS: 0.94; SPU-PLA: 0.95; Additional file 1: Fig. S1). We then compared overall detection rates, which were defined as the fraction of samples detected with any somatic mutation relative to all 71 samples of the same type. As Fig. 3A indicates, TIS showed significantly higher rate at 95.8% than PLA (70.4%) and SPU (67.6%), while the latter two were comparable (p = 0.86). Detection further increased to 85.7% (60/71) after merging mutation profiles from SPU and PLA, suggesting utility of combining liquid biopsies when economic conditions allow.

Next, we examined the association between detection rate in SPU with 14 clinicohistologic features. Additional file 1: Table S1 summarizes the resulting subgroups-specific rates and statistical significance. Detection rate did not appear to vary with the method of sample collection, as spontaneous (61.5%, 8/13) and induced sputum samples (69.0%, 40/58) resulted in similar detection rates (p = 0.74). Notably, stratification by histologic subtype or location of lung tumor led to significantly different detection rates in the resulting subgroups, including centrally vs. peripherally located tumors (79.5% vs. 53.6%, p = 0.03; Fig. 3B) and LUSC vs. LUAD (93.3% vs. 60.7%, p = 0.03; Fig. 3C). There was also a trend toward higher detection rate in smokers than in non-smokers (78.8% vs. 57.9%, p = 0.08; Fig. 3D), which was consistent with a previous study [3]. Interestingly, detection rate was lower in patients presenting with pleurisy (52.6%) than those without (77.1%), although the difference was not significant (p = 0.08).

We then compared the mutation detection performance of SPU and PLA in different patient populations (Table S1). In the 39 patients with centrally located lung tumor, overall detection from all three biopsy types rose to 94.9% for TIS, 79.5% for SPU, and 61.5% for PLA (Fig. 3E). Notably, SPU outperformed PLA in this subgroup despite a lower rate in the entire cohort. Conversely, detection rate for SPU in patients with peripherally located tumors was significantly lower than that in patients with centrally located tumors (53.6% vs. 79.5%, p = 0.03) and that for PLA samples in the same patients (53.6% vs. 85.7%, p = 0.02; Fig. 3F). Similar trends were observed in the 33 smokers, among whom detection improved for all biopsy types, and detection rate was higher in SPU (78.8) than in PLA (72.7%) albeit by a smaller margin (Fig. 3G). Similarly, SPU (93.3%) was comparable to PLA (86.7%) in detection rate in LUSC patients (Fig. 3H) and was significantly higher than SPU in LUAD patients (60.7%, p = 0.03).

We also compared overall concordance of SPU and PLA when each was compared against TIS (i.e. the reference set). Concordance rate was defined as the number of patients whose matched samples either had no alteration or shared at least one alteration divided by cohort or subgroup size. Similar to detection rate, overall concordance was comparable between the two liquid biopsy types (SPU: 67.6%, 48/71, PLA: 73.2%, 52/71, p = 0.58; Additional file 1: Table S2). Also, concordance in SPU increased in patients with LUSC (93.3%, 14/15), centrally located tumor (76.9%, 30/39), or a smoking history (75.8%, 25/33; Table 2). Consistent with increased mutation detection rate, merging SPU and PLA mutation profiles also resulted higher concordance (88.7%, 63/71).

**Table 2:** Key performance indices for detection of alterations in classic oncogenic drivers in NSCLC

Patient group	Genomic alterations	Sensitivity (%)		PPV (%)		Specificity (%)	
		SPU	PLA	SPU	PLA	SPU	PLA
Entire cohort (n = 71)	Drivers	50.0	63.5	96.3	97.1	99.8	99.8
	*EGFR*	25.0	70.8	100	100	100	100
	*KRAS*	83.3	75.0	100	100	100	100
	*ALK*	75.0	37.5	85.7	100	98.5	100
	*ROS1*	66.7	66.7	100	100.0	100	100
	*BRAF*	100.0	100.0	100	50.0	100	99.0
	*MET*	0.0	0.0	na	na	100	100
	*RET*	100	100	100	100	100	100
Centrally located lung tumors (n = 39)	Drivers	63.0	59.3	94.4	100	99.5	100
	*EGFR*	25.0	75.0	100	100	100	100
	*KRAS*	85.7	57.1	100	100	100	100
	*ALK*	71.4	28.6	83.3	100	97.1	100
	*ROS1*	66.7	66.7	100	100	100	100
	*BRAF*	100	100	100	100	100	100
	*MET*	nd	nd	nd	nd	nd	nd
	*RET*	100	100	100	100	100	100
Smokers (n = 33)	Drivers	68.8	68.8	100	100	100	100
	*EGFR*	33.3	66.7	100	100	100	100
	*KRAS*	75.0	75.0	100	100	100	100
	*ALK*	100	0.0	100	na	100	100
	*ROS1*	100	100	100	100	100	100
	*BRAF*	100	100	100	100	100	100
	*MET*	0.0	0.0	na	na	100	100
	*RET*	nd	nd	nd	nd	nd	nd
LUSC (n = 15)	Drivers	100	100	100	100	100	100
	*EGFR*	100	100	100	100	100	100
	*KRAS*	nd	nd	nd	nd	nd	nd
	*ALK*	100	100	100	100	100	100
	*ROS1*	nd	nd	nd	nd	nd	nd
	*BRAF*	nd	nd	nd	nd	nd	nd
	*MET*	nd	nd	nd	nd	nd	nd
	*RET*	nd	nd	nd	nd	nd	nd

*PLA* plasma, *PPV* positive predictive value, *SPU* sputum supernatant. All sensitivity, PPV, and specificity values are expressed in percent. na, not applicable. nd, no alteration in indicated gene detected in reference (i.e. tumor biopsy) samples

Together, the results described above showed comparable performance between sputum supernatant and plasma in three key aspects of genomic profiling in the intact cohort, including number of detected alterations, percentage of alteration-positive samples, and concordance with TIS-derived profile. Moreover, in certain subgroups, such as those with centrally located aNSCLC, sputum supernatant may be associated with greater chance of detecting genomic abnormalities.

### Performance of sputum supernatant for detecting classic NSCLC driver alterations in all patients and in specific populations

**Fig. 4:**
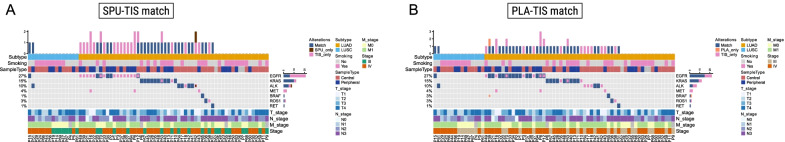
Overview of matched and unique genomic alterations in NSCLC-associated driver alterations from the **A** SPU-TIS and **B** PLA-TIS comparisons. TIS, tumor tissue. SPU, sputum supernatant. *PLA* plasma

In addition to aberrations in 520 targeted genes, we also characterized detection of clinically relevant NSCLC drivers, including those in *EGFR, KRAS, ALK, ROS1, BRAF, MET, RET,* and *NTRK*1/2/3. With TIS as reference, we compared the performance of SPU and PLA in three diagnostic metrics: sensitivity, positive predictive value (PPV), and specificity (Table 2). Figure 4 presents respective overviews of the SPU-TIS and PLA-TIS matchings. Overall sensitivity for detecting drivers was lower with SPU (50.0%) than with PLA (63.5%), while both biopsy types showed high specificity and PPVs (Table 2). Stratification by detection rate-associated clinicohistologic features revealed performance boost in some subgroups for one or both liquid biopsy types. In patients with centrally located tumors, sensitivity for SPU rose markedly to 63.0%, corresponding to 16 alterations, whereas sensitivity for PLA dropped slightly to 59.3% (14 alterations; Fig. 4A). As for smokers, SPU and PLA resulted in highly similar matches with TIS samples, leading to the same levels for the three performance indices: 68.8% sensitivity, 100% PPV and specificity (Table 2, Fig. 4). Sensitivity for both types increased from 50.0% in the unstratified cohort to 100% for LUSC patients, although there were only two alterations detected from two patients. Zooming in on each altered gene, we found a markedly lower sensitivity for SPU samples in detecting *EGFR* sensitizing mutations compared with PLA in the unstratified cohort (25.0% for SPU vs. 70.8% for PLA) or in patients with centrally located tumors (25.0% vs 75.0%) or smoking history (33.3% vs 66.7%). In contrast, *ALK* rearrangement and *KRAS* hotspot mutations were more likely to be detected from SPU cfDNA compared with PLA in all patients (*ALK*: 75.0% vs 37.5%, *KRAS*: 83.3% vs. 75.0%) and in those with centrally located tumors (*ALK*: 71.4% vs 28.6%; *KRAS*: 85.7% vs. 57.1%; Table 2). To summarize, when matched against driver alterations detected from tumor tissue samples, SPU and PLA showed high specificity (range 97.1–100%) and predominantly high PPV (range 87.5–100%). In terms of sensitivity, despite a lower level for SPU in the entire cohort, the two liquid biopsies may show comparable performance in some subgroups, including patients with centrally located tumors, smoking history, or LUSC. Also, sputum supernatant may result in more sensitive detection of certain drivers such as *ALK* rearrangements and *KRAS* hotspot mutations.

### Nomogram models for the choice of liquid biopsy for genome profiling

**Fig. 5:**
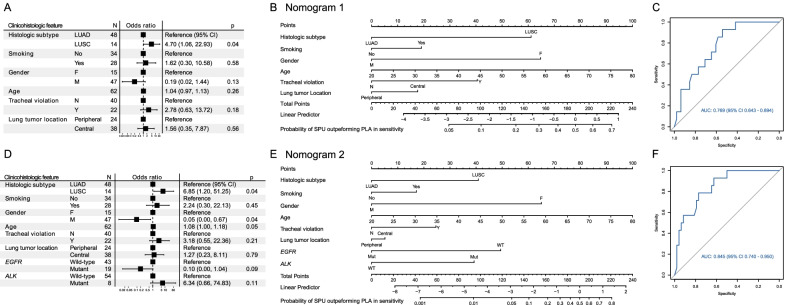
Nomograms for predicting the probability of achieving more sensitive genome profiling with sputum supernatant than with plasma based on clinicohistologic features with **A**–**C** unknown or **D**–**F** known mutation status of *EGFR* or *ALK*. **A**, **D** Forest plots of multivariate analysis for clinicohistologic characteristics associated with the probability of greater sensitivity with SPU than with PLA. **B**, **E** Nomograms constructed with features shown in (**A** and **D**). To use the nomograms, vertical lines connecting the values of each variable with the point score are first drawn at the top of the diagram. The scores for each variable are then summed to give a total points score, which is plotted along the “total points” line near the bottom. A vertical line drawn downward through scale at the bottom allows calculation of the probabilities of more sensitive genome profiling with sputum supernatant than with plasma. A worked example of (**B**) can be found in Additional file 1: Fig. S2. **C**, **F** Receiver operating characteristic analysis for validating the discrimination power of the nomograms. *AUC* area under curve, *CI* confidence interval, *SPU* sputum supernatant, *PLA* plasma

Next, we used logistic regression to construct nomograms that estimated how likely SPU would enable more sensitive detection of genomic alterations than PLA. A series of nomograms were built, taking as input varying combinations of available clinicohistologic features and were assessed by area under curve (AUC) of the receiver operating characteristic curve. Using stepwise selection (stepAIC), we arrived at two models with the greatest AUCs for clinical scenarios in which mutation status of *EGFR* and *ALK* was known (Fig. 5A–C) or unknown (Fig. 5D–F), respectively. Histologic subtype was the predominant determinant of the probability of SPU superiority. Specifically, LUSC was the only significant feature associated with a greater odds ratio (OR) for higher sensitivity with SPU (Fig. 5A, D). Tracheal violation showed a trend towards positive association with SUP superiority in both models and the male sex the opposite trend. Smoking history and centrally located tumor appeared to favor choice of SPU, although the corresponding ORs all showed wide confidence intervals that severely impaired statistical significance.

The corresponding nomograms facilitated predicting the possibility of achieving more sensitive next-generation sequencing with SPU than with PLA when the mutational status of *EGFR* and *ALK* are unknown (Nomogram 1; Fig. 5B) or known (Nomogram 2; Fig. 5E and Additional file 1: Fig. S2). Receiver operating characteristic analysis of the models demonstrated respective areas under curve (AUCs) of 0.769 (95% confidence interval [CI] 0.643–0.894; Fig. 5C) and 0.845 (95% CI 0.740–0.950; Fig. 5D). Discriminative power of the nomograms was further internally validated with 100 repetitions of tenfold cross-validation, which resulted median AUCs of 0.750 (mean 0.747, 95% CI 0.426–1.000) and 0.800 (mean 0.774, 95% CI 0.440–1.000; Additional file 1: Fig. S3).

## Discussion

In this study, we characterized the performance of sputum for genomic profiling for treatment-naïve aNSCLC patients in a number of aspects, including quality of targeted sequencing, TMB estimation, rate of alteration-positive samples and concordance with tissue-derived results, detection of driver alterations, and association between increased genome profiling sensitivity and clinicohistologic characteristics. TMB levels derived from SPU were highly correlated with those from PLA or TIS (Additional file 1: Fig. S1). Moreover, key sequencing QC metrics, number of detected alterations, detection rate, concordance with TIS-derived profiles, were comparable between SPU and PLA (Figs. 1, 2, 3, Tables 2 and Additional file 1: Fig. S1). In terms of sensitivity in detecting classic NSCLC driver alterations, SPU and PLA both achieved generally very high PPV and specificity, while sensitivity was lower with SPU than PLA in the intact cohort. However, there were specific clinicohistologic features or driver status for which SPU showed improved sensitivity to comparable or even higher levels than PLA, such as in patients with LUSC, centrally located lung tumor, or smoking history, or for altered *ALK* or *KRAS*. Finally, nomograms were constructed to predict the liquid biopsy type to enable more sensitivity detection based on common clinicohistologic characteristics (Fig. 5).

This study corroborated some findings from the one previous investigation on SPU for targeting sequencing in aNSCLC, including comparable concordance rate between SPU and PLA [3]. However, there were certain inconsistent findings. For instance, mutated *EGFR* was detected in 56% PLA and 45% SPU samples in the previous report [3], which contrasted with the greater margin we observed between PLA (70.8%) and SPU (25.0%). This greater difference could be attributed to several factors, including the definition of relevant alterations in *EGFR* and experimental protocol. An extra step of pancreatin-catalyzed mucolysis was performed in our study, which could have led to loss of DNA and hence fragments harboring mutant *EGFR*. Indeed, sensitivity in *EGFR* mutation detection appeared positively associated with amount of extracted DNA [5]. In a report on a PCR-based genotyping approach, an improved mucolytic solution led to an approximately 16-fold increase in DNA yield than the comparator, achieving a mean yield of 3898 ng from 2 ml sputum (2211 ng in this study). As expected, sensitivity for *EGFR* mutation detection increased from 50 to 75% after improved mucolysis in 40 LUAD cases. Despite the technical differences between targeted sequencing and PCR, these results suggested that genomic profiling with SPU may also benefit from increasing input SPU volume and a better DNA extraction method.

Based clinicohistologic features associated with improved detection from SPU, we also constructed two nomograms to help choose between SPU and PLA for targeted sequencing. Typical clinical scenarios for these models include before molecular testing for treatment-naïve patients (Nomogram 1) and after progression on therapy targeting (Nomogram 2). For instance, according to Nomogram 2, a 60-year-old, female, *EGFR*-positive and *ALK*-negative smoker with centrally located LUSC that has violated the trachea would have a > 75% chance of more sensitive detection using SPU than PLA samples (Additional file 1: Fig. S2). Thus, these nomograms may facilitate choice of liquid biopsy for genomic profiling for clinicians. To our knowledge, our report is the first to propose a quantitative approach for such purpose. Compared with plasma, sputum represents a truly non-invasive biopsy with easy collection protocol, which may be developed into standard procedures that do not require healthcare professionals and therefore encourage patient adherence and reduce the duration and frequency of hospital visits. This extra advantage could be especially relevant amidst the current COVID-19 pandemic.

In addition to histopathologic work-up, sputum cytology has been an long-established approach for lung cancer diagnosis and staging [10]. The study on PCR-based *EGFR* genotyping categorized samples into three groups based on cytologic evaluation of the precipitates after cfDNA extraction [5, 11] and found significantly improved sensitivity for detecting *EGFR* mutations from SPU with accompanying malignant cells-positive precipitates compared with the other subgroups (100% vs. 71.4% and 0% in the other two groups) [5]. Therefore, cytology review could play a similar role to histologic confirmation of tumor content for tumor tissue sequencing. Therefore, the predictive power of our nomograms could be benefit from integrating characterization of the precipitate by either conventional cytologic review or novel, single-cell approach based on isolated exfoliated tumor cells [12].

We also established two nomograms based clinicohistologic features and mutational status of *EGFR* and *ALK* for predicting the relative performance of SPU and PLA in molecular testing. Both models enabled moderate to borderline high classification accuracy as indicated by AUC values of 0.75–0.85, and Nanogram 2 had a greater AUC than Nanogram 1 (respective AUCs: 0.769 and 0.845; Fig. 5). Accordingly, if an *EGFR*- or *ALK*-positive patient has acquired resistance on cognate targeted therapy, knowledge their status could further help choose a liquid biopsy with greater performance. Consistent with their associations with sensitivity in SPU, *EGFR* positivity favored PLA while *ALK* positivity favored SPU. However, external validation of the models is warranted to further characterize their clinical value.

This study suffers from several limitations. First, despite enrolling 71 patients, subgrouping by clinicohistologic features reduced the subgroup size, thereby lowering the statistical power to detect significant associations. In addition, the two nomogram models need validation with large external cohorts. Also, Nomogram 2 was intended for as alteration-gnostic use but was built on results from patients with no prior molecular testing. As genetic heterogeneity is known to increase after emergence of therapeutic resistance [13–15], Nomogram 2 may be improved by using genomic profiles of patients who have progressed on targeted therapy. Furthermore, efforts are needed to elucidate whether sputum cfDNA is more likely to be limited to intrathoracic tumors or may come from multiple disease sites, which could further help differentiate the clinical application for sputum and blood biopsies [16, 17].

## Conclusion

In summary, we investigated the utility of sputum as an alternative liquid biopsy to plasma for genomic profiling for aNSCLC patients and, for the first time, constructed nomograms for choice of biopsy type based on common clinicohistologic characteristics. Future research on previously treated patients and incorporating cytologic review and improved mucolysis protocol is therefore warranted to further characterize the clinical utility of sputum aNSCLC.

## Supplementary Information

**Additional file 1: Figure S1.** Tumor mutation burden estimates were highly correlated between (A) tumor tissue and sputum supernatant, (B) tumor tissue and plasma, and (C) sputum supernatant and plasma. Shown on each plot are the corresponding Pearson correlation coefficients and *p*-value. **Figure S2.** A worked example of how to use the nomogram to predict the probability achieving more sensitive genome profiling with sputum supernatant (SPU) than with plasma (PLA). For a 60-year-old female smoker with *EGFR*-positive, *ALK*-wild-type centrally located lung tumor that has violated the trachea, the points for each risk factor add up to 219 (blue, dashed lines). A vertical line (red, solid) is then drawn from 219 on the “Total points” line down (third to the last) to the last line to predict likelihood of SPU outperforming PLA in sensitivity (77%). **Figure S3.** Areas under curve (AUCs) resulting from discrimination power evaluation of Nomograms 1 and 2 with 10-fold cross-validation, revealing significantly higher AUCs for Nomogram 2. **Table S1.** Mutation detection rates using sputum supernatant in different patient populations. **Table S2.** Overall concordance rates resulting of mutation matching between tissue-sputum or tissue-plasma in the entire cohort or selected patient subgroups.

## Data Availability

The datasets used and/or analyzed during the current study are available from the corresponding author (doctorzcz@163.com) on reasonable request.
