# Supplementary material for: Comprehensive characterization reveals sputum supernatant as a valuable alternative liquid biopsy for genome profiling in advanced non-small cell lung cancer
Source: Respir Res. 2022 Jul 1;23:175. doi: 10.1186/s12931-022-02097-4 (PMC9247971; doi:10.1186/s12931-022-02097-4)
Supplement: Supplementary file 1 — Additional file 1: Figure S1. Tumor mutation burden estimates were highly correlated between (A) tumor tissue and sputum supernatant, (B) tumor tissue and plasma, and (C) sputum supernatant and plasma. Shown on each plot are the corresponding Pearson correlation coefficients and p-value. Figure S2. A worked example of how to use the nomogram to predict the probability achieving more sensitive genome profiling with sputum supernatant (SPU) than with plasma (PLA). For a 60-year-old female smoker with EGFR-positive, ALK-wild-type centrally located lung tumor that has violated the trachea, the points for each risk factor add up to 219 (blue, dashed lines). A vertical line (red, solid) is then drawn from 219 on the “Total points” line down (third to the last) to the last line to predict likelihood of SPU outperforming PLA in sensitivity (77%). Figure S3. Areas under curve (AUCs) resulting from discrimination power evaluation of Nomograms 1 and 2 with 10-fold cross-validation, revealing significantly higher AUCs for Nomogram 2. Table S1. Mutation detection rates using sputum supernatant in different patient populations. Table S2. Overall concordance rates resulting of mutation matching between tissue-sputum or tissue-plasma in the entire cohort or selected patient subgroups. [file 12931_2022_2097_MOESM1_ESM.docx]

**Additional figure legends**

Figure S1. Tumor mutation burden estimates were highly correlated between (A) tumor tissue and sputum supernatant, (B) tumor tissue and plasma, and (C) sputum supernatant and plasma. Shown on each plot are the corresponding Pearson correlation coefficients and *p*-value.

**
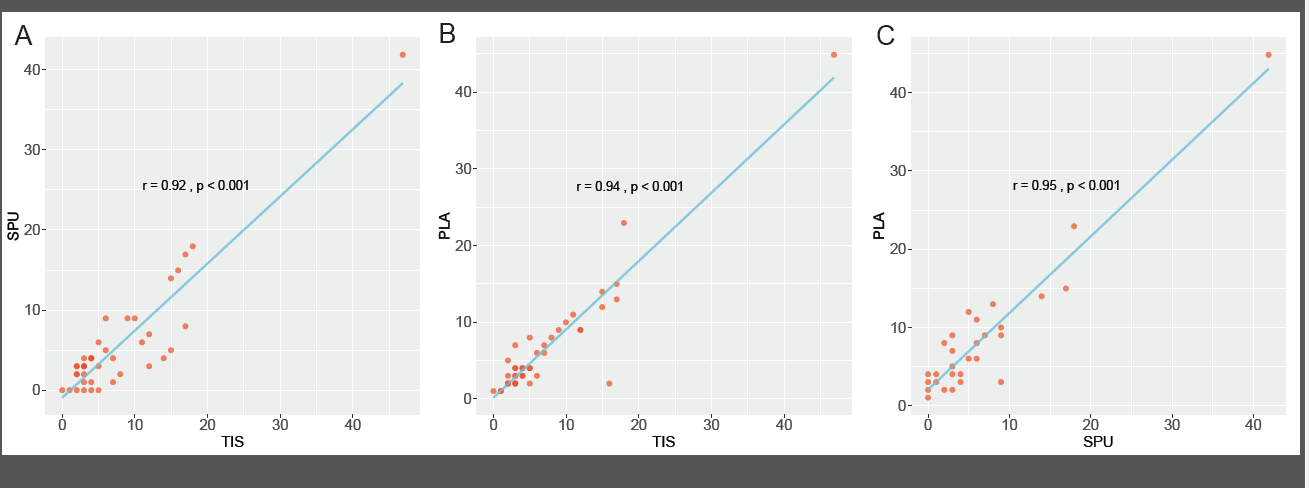
**

Figure S2. A worked example of how to use the nomogram to predict the probability achieving more sensitive genome profiling with sputum supernatant (SPU) than with plasma (PLA). For a 60-year-old female smoker with *EGFR*-positive, *ALK*-wild-type centrally located lung tumor that has violated the trachea, The points for each risk factor add up to 219 (blue, dashed lines). A vertical line (red, solid) is then drawn from 219 on the “Total points” line down (third to the last) to the last line to predict likelihood of SPU outperforming PLA in sensitivity (77%).


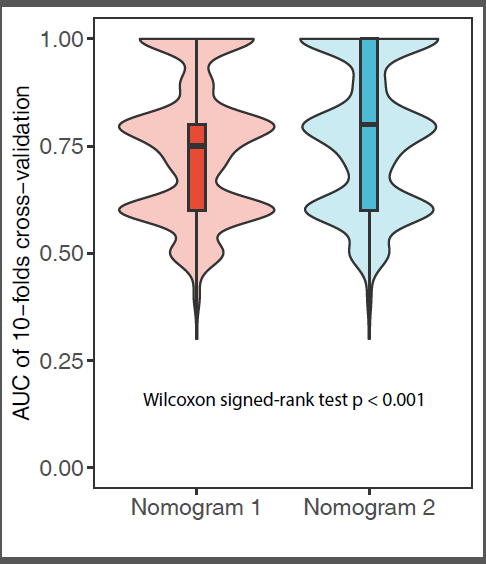

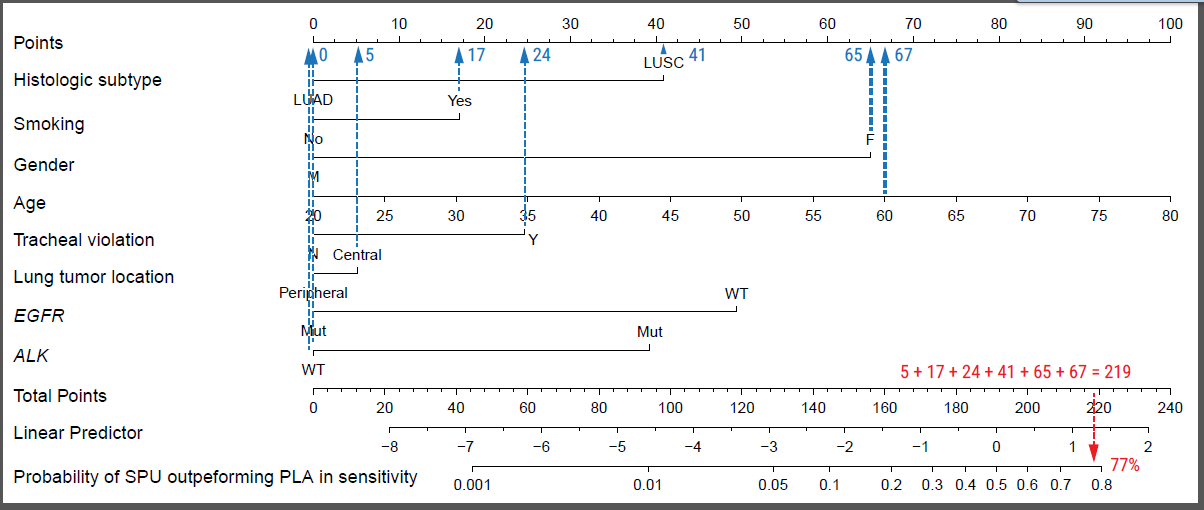


Figure S3. Areas under curve (AUCs) resulting from discrimination power evaluation of Nomograms 1 and 2 with 10-fold cross-validation, revealing significantly higher AUCs for Nomogram 2.

**Additional tables**

Table S1. Mutation detection rates using sputum supernatant in different patient populations.

|  |  | **SPU** | | **PLA** | | ***p* (SPU vs. PLA)** |
| --- | --- | --- | --- | --- | --- | --- |
| **Clinicohistologic feature** | | % (n) | *p* | % (n) | *p* |  |
| Sex | Female | 56.2 (9/16) | 0.37 | 56.2 (9/16) | 0.22 | 1.00 |
|  | Male | 70.4 (38/54) |  | 74.1 (40/54) |  |  |
| Histologic subtype | LUAD | 60.7 (34/56) | 0.03 | 66.1 (37/56) | 0.20 | 0.82 |
|  | LUSC | 93.3 (14/15) |  | 86.7 (13/15) |  |  |
| Smoking | Smoker | 78.8 (26/33) | 0.08 | 72.7 (24/33) | 0.80 | 0.55 |
|  | Non-smoker | 57.9 (22/38) |  | 68.4 (26/38) |  |  |
| Location of lung tumor | Central | 79.5 (31/39) | 0.03 | 61.5 (24/39) | 0.05 | 0.10 |
|  | Peripheral | 53.6 (15/28) |  | 85.7 (24/28) |  |  |
| Stage | III | 68.2 (15/22) | 1.00 | 72.7 (16/22) | 1.00 | 1.00 |
|  | IV | 68.8 (33/48) |  | 70.8 (34/48) |  |  |
| Sputum type | Induced | 61.5 (8/13) | 0.74 | 61.5 (8/13) | 0.51 | 1.00 |
|  | Non-induced | 69.0 (40/58) |  | 72.4 (42/58) |  |  |
| M stage | M0 | 73.7 (14/19) | 0.77 | 68.4 (13/19) | 1.00 | 1.00 |
|  | M1 | 68.8 (33/48) |  | 70.8 (34/48) |  |  |
| Nodal metastasis | Yes | 67.6 (46/68) | 1.00 | 72.1 (49/68) | 0.49 | 0.61 |
|  | No | 100 (2/2) |  | 50.0(1/2) |  |  |
| Brain metastasis | Yes | 83.3 (10/12) | 0.32 | 66.7 (8/12) | 0.73 | 0.61 |
|  | No | 65.5 (38/58) |  | 72.4 (42/58) |  |  |
| Pleural metastasis | Yes | 66.7 (18/27) | 0.80 | 74.1 (20/27) | 0.79 | 0.84 |
|  | No | 69.8 (30/43) |  | 69.8 (30/43) |  |  |
| Bone metastasis | Yes | 69.6 (16/23) | 1.00 | 78.3 (18/23) | 0.42 | 0.83 |
|  | No | 68.1 (32/47) |  | 68.1 (32/47) |  |  |
| Tracheal violation | Yes | 78.3 (18/23) | 0.28 | 69.6 (16/23) | 0.78 | 0.67 |
|  | No | 63.6 (28/44) |  | 72.7 (32/44) |  |  |
| Blood vessel invasion | Yes | 66.7 (8/12) | 1.00 | 58.3 (7/12) | 0.29 | 0.78 |
|  | No | 69.6 (39/56) |  | 75 (42/56) |  |  |
| Pleurisy | Yes | 52.6 (10/19) | 0.08 | 73.7 (14/19) | 1.00 | 0.48 |
|  | No | 77.1 (37/48) |  | 72.9 (35/48) |  |  |

All *p* values were from Fisher’s exact test. LUAD, lung adenocarcinoma. LUSC, lung squamous cell carcinoma. PLA, plasma. SPU, sputum supernatant. TIS, tumor biopsy.

Table S2. Overall concordance rates resulting of mutation matching between tissue-sputum or tissue-plasma in the entire cohort or selected patient subgroups.

| **Patients** | **Concordance rate, n (%)** | | ***p*** |
| --- | --- | --- | --- |
|  | SPU-TIS | PLA-TIS |  |
| **Entire cohort** | 48 (67.6) | 52 (73.2) | 0.58 |
| **Location of lung tumor** | | | |
| Central (n = 39) | 30 (76.9) | 26 (66.7) | 0.45 |
| Peripherally (n = 28) | 16 (57.1) | 24 (85.7) | 0.04 |
| **Smoking history** | | | |
| Smokers (n = 33) | 25 (75.8) | 24 (72.7) | 1.00 |
| Non-smokers (n = 38) | 23 (60.5) | 28 (73.7) | 0.33 |
| **Histologic subtype** | | | |
| LUAD (n = 56) | 34 (60.7) | 40 (71.4) | 0.32 |
| LUSC (n = 15) | 14 (93.3) | 12 (80.0) | 0.60 |

All *p* values were from Fisher’s exact test. Concordance rate was defined as the fraction of the total number of true positive patients (carrying at least one alteration that was detected from both matched samples) and true negative patients (no mutation detected from either sample) relative to the entire cohort or indicated subgroup. LUAD, lung adenocarcinoma. LUSC, lung squamous cell carcinoma. PLA, plasma. SPU, sputum supernatant. TIS, tumor biopsy.
